# Setting the bar

**DOI:** 10.7554/eLife.39068

**Published:** 2018-07-17

**Authors:** Charles Y Feigin, Ricardo Mallarino

**Affiliations:** Department of Molecular BiologyPrinceton UniversityPrincetonUnited States

**Keywords:** *Columba livia*, pigeon, introgression, NDP, pigmentation, phenotypic diversity, Other

## Abstract

Analyzing the genomes of rock pigeons demonstrates that genetic variation comes in many forms and can have unexpected origins.

**Related research article** Vickrey AI, Bruders R, Kronenberg Z, Mackey E, Bohlender RJ, Maclary ET, Maynez R, Osborne EJ, Johnson KP, Huff CD, Yandell M, Shapiro MD. 2018. Introgression of regulatory alleles and a missense coding mutation drive plumage pattern diversity in the rock pigeon. *eLife* **7**:e34803. doi: 10.7554/eLife.34803

Rock pigeons (*Columba livia*) are an immensely successful species, having spread around the world through their association with humans. One of the secrets to their success is their impressive phenotypic diversity, which has helped them to adapt to different environments and made them a historical favorite of bird fanciers, including Darwin. Indeed, in *On the Origin of Species*, the great naturalist wrote: "a score of pigeons might be chosen, which if shown to an ornithologist, and he were told that they were wild birds, would certainly, I think, be ranked by him as well-defined species".

Darwin capitalized on the remarkable diversity of rock pigeons to describe concepts like artificial selection, thus catapulting this species into the spotlight. However, although the rock pigeon has long been an exemplar of how selection pressures and variation can produce phenotypic novelty, biologists have only recently begun to unravel the genetic underpinnings of their stunning diversity (Shapiro et al., 2013; Domyan and Shapiro, 2017).

Color patterns – like those found in the wings of rock pigeons – are among the most variable and conspicuous traits found nature, and they play key roles in the survival and reproduction of individuals in the wild (Cuthill et al., 2017). Like all developmental processes, the mechanisms that drive color patterning must translate inherited information in the genome into spatial information in an organism to produce consistent phenotypes. Many of the key genes involved in coloring and pigmentation are known (Barsh, 1996; Mills and Patterson, 2009), but much less is known about how these genes are regulated. Now, in eLife, Michael Shapiro of the University of Utah and colleagues – including Anna Vickrey as first author – report surprising new insights into color patterning in rock pigeons (Vickrey et al., 2018).

They started by scanning the genomes of multiple pigeons possessing one of the four primary color patterns on their wings: t-check, checker, bar and barless (Figure 1A). They identified a region of the genome that was associated with the two colored or melanistic patterns (t-check and checker), and found that this region corresponded with a region called the *C* locus: classical breeding experiments had previously shown that this region was involved in color patterning. All the individual pigeons with melanistic phenotypes were also found to carry at least one variant copy of this region.

**Figure 1.**
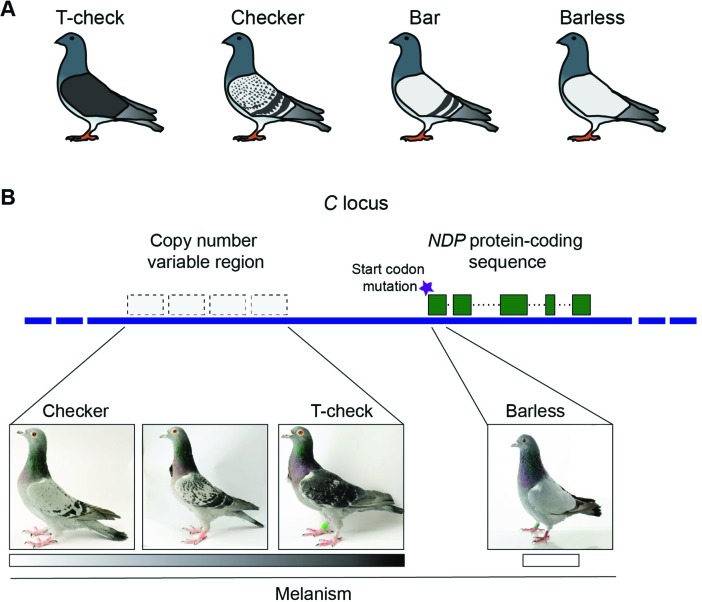
**Genetic origins of different phenotypes in rock pigeons** (*Columba livia*). (**A**) Rock pigeons can have one of four patterns on their wings: t-check (which is the most melanistic), checker, bar and barless (which is the least melanistic). (**B**) The genomic region that controls wing pattern in pigeons, dubbed the *C* locus, harbors a copy number variable (CNV) region that contains between one and four repeated units per chromosome. Increased copy number was associated with higher levels of melanism (shown by the grayscale): this suggests that the CNV region contains an element that regulates the expression of a gene called *NDP*. In addition, a mutation (purple star) in the start codon for this gene is likely responsible for the barless phenotype. Note: the unlabeled pigeon is an intermediate between the checker and t-check phenotypes. Images courtesy of the Genetic Science Learning Center, University of Utah; www.learn.genetics.utah.edu.

Next Vickrey et al. – who are based at Utah, the University of Texas, Houston and the University of Illinois Urbana-Champaign – identified a copy number variable (CNV) region within the *C* locus. The number of copies and the level of melanism were strongly correlated (Figure 1B), but some birds displayed melanistic patterns without any associated increase in copy number. Thus, while it appears that the checker variant of the *C* locus must be present in order for melanistic patterns to be produced, increased copy number is not essential.

Digging deeper, Vickrey et al. found that the CNV region was also associated with the expression level of a nearby gene called *NDP*, which has previously been linked to pigmentation in crows. Unlike other neighboring genes, the expression of *NDP* was elevated in the wings of birds with more melanistic patterns. Vickrey et al. went on to show that these differences in expression are regulated by elements lying within the *C* locus, leading them to speculate that the CNV region contains an *NDP* enhancer that promotes melanistic phenotypes.

But this is not the whole story. Regulatory changes do not explain the barless phenotype. Instead, Vickrey et al. found that barless birds contain a mutation in the start codon for the *NDP* gene (Figure 1B). Similar mutations in humans are associated with a form of hereditary blindness. Consistent with this, many barless birds have impaired vision. Moreover, the defective *NDP* allele is rarely observed outside of captivity. In domestic animals, however, alleles associated with disease states can persist because of strong artificial selection: what flies in captivity doesn’t always fly in nature! By studying a series of alleles at a single locus, Vickrey et al. provide evidence that patterning phenotypes can be generated by both regulatory changes and protein coding changes at a single locus, which is a remarkable finding.

In Darwin’s discourse on pigeons, he posits that domestic pigeons originated from a single species, the wild pigeon. However, pigeon fanciers after Darwin’s time suspected that this was not the case, as the checker wing pattern seen in rock pigeons looks very similar to that seen in the African speckled pigeon (*C. guinea*).

Armed with genomic tools, Vickrey et al. set out to settle this matter. They compared the genomes of the rock pigeon and the African speckled pigeon, and found that most of the regions in these genomes displayed the levels of differentiation you would expect to see in two species that split 4–5 million years ago. However, when they compared the sequences from the *C* locus, they found very few differences. Remarkably, the sequences of rock pigeons carrying the checker allele were more similar to the sequences of speckled pigeons than they were to the corresponding regions of rock pigeons carrying the bar allele. Through additional tests, Vickrey et al. were able to establish that that the checker haplotype likely originated in African speckled pigeon and was acquired by the rock pigeon through introgression – that is, by the transfer of genetic material between hybridizing species.

Why wasn’t the *NDP* allele pruned out of the rock pigeon genome, like is the case with most foreign alleles? Vickrey et al. believe that the answer to this question lies in the fact that *NDP* has been linked to various reproductive and physiological traits. Thus, even though the changes in wing color pattern are the most conspicuous effects of the introgression, the simultaneous transfer of other beneficial traits may be exerting strong selective pressures to maintain the allele in the population. Introgression is increasingly recognized as a critical source of genetic variation for species experiencing selective pressures (Martin and Jiggins, 2017): in particular, introgression means that the rate at which mutation and/or standing variation can promote phenotypic diversity is less of a limiting factor.

By characterizing the molecular basis and evolutionary history of wing color pattern diversity in pigeons, Vickrey et al. have made significant headway in our understanding of the origin and maintenance of phenotypic diversity. However, many key questions remain unanswered. How is information encoded in the *C* locus rendered into such different color patterns in the wing? How do modifiers of the checker variant affect the relationship between CNV copy number and degree of melanism? Moving forward, functional analyses will be essential to address these mechanistic questions, and with a powerful model system like the pigeon, Vickrey, Shapiro and colleagues are ready to rock.
